# Evaluation of the “Shifting Weight using Intermittent Fasting in night-shift workers” weight loss interventions: a mixed-methods protocol

**DOI:** 10.3389/fpubh.2023.1228628

**Published:** 2023-09-07

**Authors:** Corinne Davis, Maxine P. Bonham, Sue Kleve, Jillian Dorrian, Catherine E. Huggins

**Affiliations:** ^1^Department of Nutrition, Dietetics and Food, Monash University, Notting Hill, VIC, Australia; ^2^UniSA Justice & Society, University of South Australia, Adelaide, SA, Australia; ^3^Deakin University, Geelong, Australia, Institute for Health Transformation, Global Centre for Preventive Health and Nutrition, School of Health and Social Development, Faculty of Health, Geelong, VIC, Australia

**Keywords:** obesity, over-weight, nutrition, diet, weight-management, shift work, night work

## Abstract

**Introduction:**

Shift workers are at a greater risk for obesity-related conditions. The impacts of working at night presents a challenge for designing effective dietary weight-loss interventions for this population group. The Shifting Weight using Intermittent Fasting in night-shift workers (SWIFt) study is a world-first, randomized controlled trial that compares three weight-loss interventions. While the trial will evaluate the effectiveness of weight-loss outcomes, this mixed-methods evaluation aims to explore for who weight-loss outcomes are achieved and what factors (intervention features, individual, social, organisational and wider environmental) contribute to this.

**Methods:**

A convergent, mixed-methods evaluation design was chosen where quantitative and qualitative data collection occurs concurrently, analyzed separately, and converged in a final synthesis. Quantitative measures include participant engagement assessed via: dietary consult attendance, fulfillment of dietary goals, dietary energy intake, adherence to self-monitoring, and rates for participant drop-out; analyzed for frequency and proportions. Regression models will determine associations between engagement measures, participant characteristics (sex, age, ethnicity, occupation, shift type, night-shifts per week, years in night shift), intervention group, and weight change. Qualitative measures include semi-structured interviews with participants at baseline, 24-weeks, and 18-months, and fortnightly audio-diaries during the 24-week intervention. Interviews/diaries will be transcribed verbatim and analyzed using five-step thematic framework analysis in NVivo. Results from the quantitative and qualitative data will be integrated via table and narrative form to interrogate the validity of conclusions.

**Discussion:**

The SWIFt study is a world-first trial that compares the *effectiveness* of three weight-loss interventions for night shift workers. This mixed-methods evaluation aims to further explore the effectiveness of the interventions. The evaluation will determine for who the SWIFt interventions work best for, what intervention features are important, and what external factors need to be addressed to strengthen an approach. The findings will be useful for tailoring future scalability of dietary weight-loss interventions for night-shift workers.

**Clinical trial registration:** This evaluation is based on the SWIFt trial registered with the Australian New Zealand Clinical Trials Registry [ACTRN 12619001035112].

## 1. Introduction

Shift workers make up an almost 30% subset of the workforce worldwide, who undertake critical work for a 24-h society to function (1). This essential work comes with a disproportionately greater risk for obesity, type 2 diabetes, and cardiovascular disease (2, 3). Night-shift workers fall within the highest risk category, with greater odds for these poor health outcomes (2, 3). Given the link between increased weight and metabolic conditions (4–6), weight loss is a logical target for reducing disease risk in this population. There is currently limited guidance for night-shift workers on best-practice dietary approaches for weight loss (7). Recent reviews on nutrition or weight-loss interventions for night-shift workers have identified a limited number of published studies and no statistically or clinically significant effect for weight loss (8). More research is needed to understand what dietary weight-loss interventions are best suited to night-shift workers (2, 9).

Night work is associated with metabolic misalignment, circadian disruption, and differences in 24-h energy expenditure compared to day workers, which in turn, is thought to contribute to weight gain (10). These factors offer a target for dietary weight loss approaches for night-shift workers. In order for weight loss interventions to be successful in the night-shift working population, meal timing may need to be considered in addition to energy restriction (10, 11). The Shifting Weight using Intermittent Fasting in night shift workers (SWIFt) study is a randomized-controlled (RCT), three-arm parallel intervention to compare the effectiveness of three dietary interventions on weight loss in night-shift workers, to investigate whether the timing of energy restriction is beneficial for night-shift workers (11). The SWIFt trial aims to investigate whether a 5:2 intermittent fasting approach that aligns two fast periods with night shift has benefits on both weight and metabolic outcomes (11). The 5:2 approach limits energy consumption to 20%–25% of energy requirements on two ‘fast’ days per week and *ad libitum* eating on the remaining 5 days (11). While the SWIFt RCT will examine the effectiveness of the dietary interventions for weight loss, it is now recommended evaluations are conducted alongside the trial to more fully understand the factors (both mechanisms of action and external influences) contributing to intervention effectiveness (12–18). This information is needed to improve effectiveness and to tailor future scalability of an intervention (12–18). Evaluations typically explore participant engagement to determine the extent to which the “active ingredients” or proposed mechanisms of action of an intervention can explain the study outcomes (13, 15). In addition, a key consideration in interventions involving human participants is *how* participants engage with the study requirements, that is, what drives participant “responsiveness” to the requirements of the intervention and leads to engagement (18). This allows a deeper understanding of what may drive participant engagement to a dietary intervention in a real-world setting.

Evaluations also typically explore whether contextual factors influence outcomes and underlying mechanisms (15). Contextual factors may be positive (enablers) or negative (barriers) and relate to different spheres of influence ranging from the individual (e.g., participant characteristics such as age or personality type), social (e.g., peer influence), and organisational (e.g., workplace environment), to the wider environment (19). An understanding of these underlying mechanisms and contextual factors in the SWIFt study will be useful to understand who can benefit the most from the interventions and to replicate the potential benefits in other non-research settings.

### 1.1. Aims and research questions

The overall aim of this study is to explore the contributing factors (intervention features, individual, social, organizational and wider environmental) to weight loss. Specifically:

- To describe participant engagement overall and for each of the SWIFt weight loss interventions.
- Explore factors (intervention features, individual, social, organisational and wider environmental) that influence participant engagement for each of the SWIFt weight loss interventions.
- Explore for who weight change outcomes are achieved for each of the SWIFt weight loss interventions, and the influence of participant engagement.

## 2. Intervention of interest

The full study protocol has been described previously (11). In summary, shift workers will be randomized to one of three dietary interventions including: 20% continuous daily energy restriction (CER), versus 5:2D (day fast twice per week), versus 5:2N (night fast twice per week aligning with a night shift) for 24-weeks with a 12-month follow-up period. In the 5:2 interventions, participants will aim to limit intake for each fasting period to 2,100 kJ/day (females) or 2,500 kJ/day (males), providing a weekly energy restriction comparable to the CER intervention (i.e., 20%). Study participants will see a study dietitian fortnightly for the first 8 weeks and then monthly for the last 16 weeks of the intervention period and will be provided with study foods for 2 days per week. The dietitian will explain the dietary intervention, set goals with participants, discuss strategies to assist with dietary adherence, and monitor progress. At the conclusion of the 24-week intervention, participants will be given practical suggestions and food options for continuing their allocated dietary intervention. Participants will be followed up at 2-, 6- and 12-months’ time during the follow-up period to monitor progress. No food will be supplied to participants during this time.

## 3. Methods and analysis

**Figure 1:**
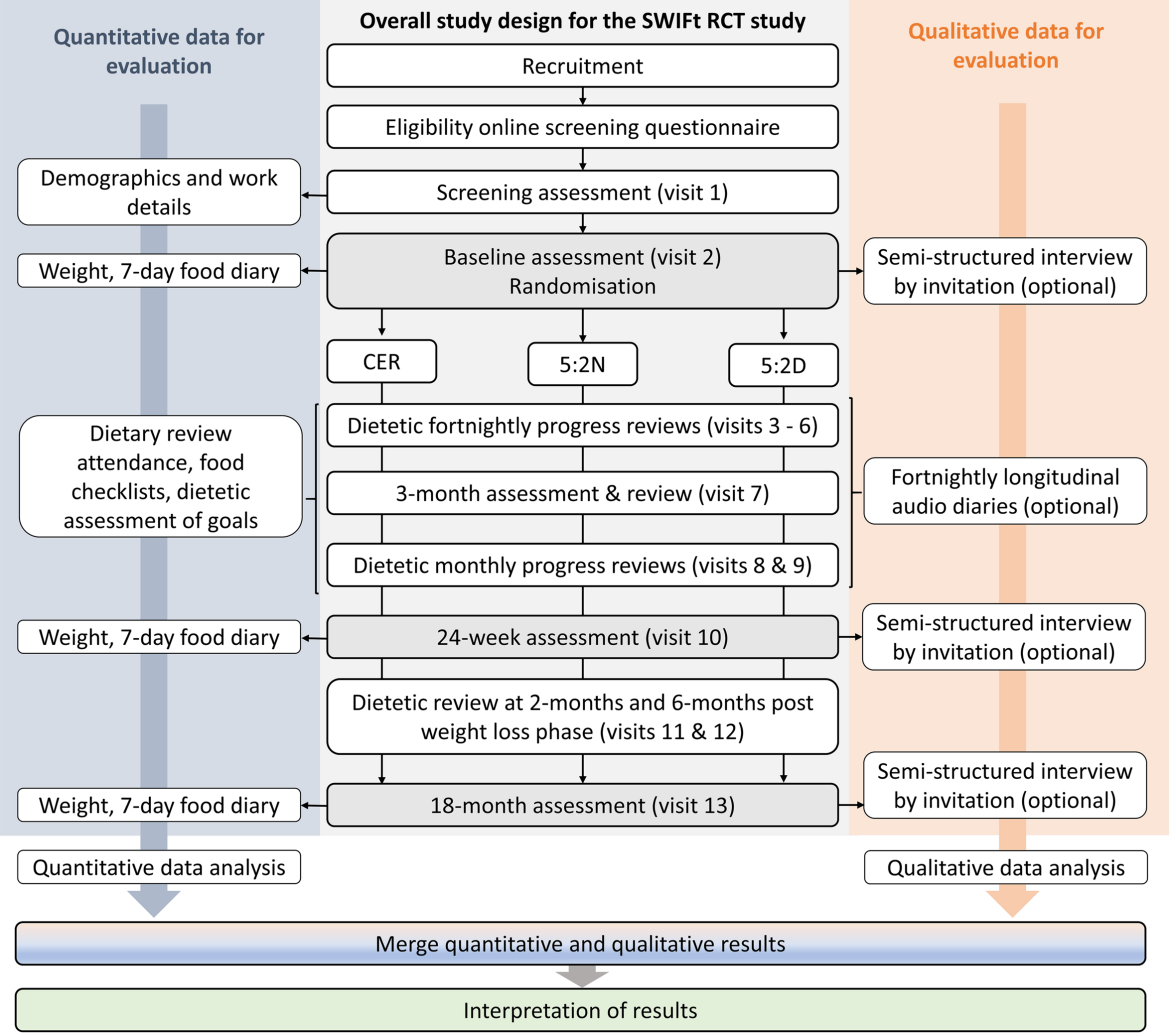
Mixed-methods evaluation design of the Shifting Weight using Intermittent Fasting in night shift workers study. RCT, randomized controlled trial; CER, continuous energy restriction; 5:2N, intermittent fasting protocol whereby the two fast days coincide with night shifts; 5:2D, intermittent fasting protocol whereby the two fast days coincide with day shifts and/or days off.

This study uses “pragmatism” as a theoretical perspective guiding research design. Pragmatism is oriented to what works and choosing multiple, best-fit methods to answer the research questions posed (20, 21). A convergent, mixed-methods, experimental (or intervention) design (20) has been selected given that the purpose of this study is to evaluate a randomized controlled trial (experimental intervention). In addition, this design has been selected due to the timeframe of the SWIFt study, where the outcomes of the first set of quantitative data analysis will not be known until all participants have completed 24-weeks of their dietary intervention (see Figure 1). As such, quantitative and qualitative data will be collected concurrently during the intervention and follow-up period of the SWIFt study. A mixed-methods approach has been chosen to obtain different but complementary data on the research aims and to bring together the strengths of both methods (20–22).

### 3.1. Participants

Eligibility criteria and recruitment for the overall SWIFt study has been previously described (11). In summary, study participants will be aged between 25 and 65 years and working a minimum of two nights per week for a minimum of six consecutive months. Participants will have a body mass index (BMI) of ≥28 kg/m^2^ for non-Asian men and women and ≥ 26 kg/m^2^ for Asian men and women and be able to attend either study site. Please refer to the previously published study protocol for exclusion criteria relating to medical and lifestyle conditions that may affect body composition, metabolism, or ability to follow the dietary protocol (11). Participants who consent to be invited to complete longitudinal audio diaries (LADs) and/or semi-structured interviews will be contacted after randomization based on a maximum-variation sampling approach (23). Maximum variation is used to provide a diverse set of participant viewpoints across age, sex, occupation, shift-type, and intervention group. Participants who discontinue with the intervention will also be invited for an interview. Identifying sample size *a priori* for qualitative research is problematic (24), therefore data collection and preliminary data analysis will occur concurrently, and recruitment will cease once maximum-variation is met, and when limited new information is identified.

### 3.2. Data sources

Key objectives and data sources used to meet the overall aim of this mixed-methods evaluation have been developed in line with similar evaluations (25) and are outlined in Table 1. Qualitative data will be collected and reported in line with the consolidated criteria for reporting qualitative research (COREQ) (26). It is anticipated that data collection will be complete by September 2023. For the purposes of this evaluation, participant “engagement” with the intervention is defined as whether participants: follow the general requirements of the study protocol (e.g., attendance of dietetic consults, self-monitoring); follow the dietary requirements of the intervention; and choose to continue with the intervention (e.g., rates of drop-out).

**Table 1:** Objectives, data sources and analysis for the evaluation of Shifting Weight using Intermittent Fasting in night shift workers (SWIFt) study.

Objective	Quantitative data source/analysis	Qualitative data source/analysis
1. To describe participant engagement overall and for each of the SWIFt weight loss interventions		
1.1 Dietary review attendance.	Number of dietetic reviews attended (Yes/No). Also described as percentage of reviews attended overall and by intervention group.	
1.2 Dietary goals.	Number of reviews where participant is following dietary goals as assessed by dietitian (Yes/No). Also described as percentage of reviews where goals are met overall and by intervention group.	
1.3 Dietary energy restriction.	Pre- and post-intervention energy restriction: 7-day food diary (baseline compared to 24-weeks and 18-months). Overall and by intervention group.	
1.4 Self-monitoring.	Number of checklists collected. Also described as percentage of food checklists collected overall and by intervention group.	
1.5 Other.	Frequency and percentage of participants who drop-out/time to drop out overall and by intervention group.	
2. Explore factors (intervention features, individual, social, organisational and wider environmental) that influence participant engagement for each of the SWIFt weight loss interventions.		
2.1 Intervention features.	Relationship between intervention group and engagement measures (1.1, 1.2, 1.3, 1.4, 1.5).	Semi-structured interviews. Longitudinal audio diaries (LADs). Features of intervention mapped to the BCT and TDF.
2.2 Contextual features.	Relationship between participant characteristics* and engagement measures (1.1, 1.2, 1.3, 1.4, 1.5). Participant reasons for drop-out.	Semi-structured interviews. Longitudinal audio diaries (LADs). Enablers/barriers to engagement mapped to the SEM.
3. Explore for who weight change outcomes are achieved for each of the SWIFt weight loss interventions and the influence of participant engagement.		
3.1 Intervention features.	Relationship between weight outcome and engagement measures (1.1, 1.2, 1.3, 1.4).	Participant interviews. Longitudinal audio diaries (LADs). Features of intervention mapped to the BCT and TDF.
3.2 Contextual features.	Relationship between participant characteristics* and weight outcome (24-weeks and 18-months).	Participant interviews. Longitudinal audio diaries (LADs). Enablers/barriers to outcomes mapped to the SEM.

^*^Participant characteristics include: age, sex, ethnicity, occupation, shift schedule, number of night shifts per fortnight; years in night shift; BCT, Behaviour Change Taxonomy; TDF, Theoretical Domains Framework; SEM, Social-Ecological Model.

#### 3.2.1. Quantitative data sources

The SWIFt study data will be entered primarily via direct data entry using Research Electronic Data Capture (REDCap; Vanderbilt University, Nashville, United States). REDCap is a secure web interface with data checks during data entry and uploading to ensure data quality and is housed on secure servers operated by Monash University, Australia.

##### 3.2.1.1. Dietary review attendance

Details of participant attendance at each dietetic consult/review will be recorded, including the following information: attendance or missed appointment, reasons for missed appointment (if known), and a summary of the discussion at the consult. Each participant will have a score out of 8 (the total number of dietetic reviews over the 24-weeks), representing the number of reviews attended. See Objective 1.1 in Table 1.

##### 3.2.1.2. Dietary goals

At each study visit, the study dietitian will judge and record (yes/no) whether the participant has followed the goals of their allocated dietary approach (e.g., followed the dietary changes recommended by the dietitian for the CER intervention, or consumed the study foods for each fasting period for the 5:2 diets). Each participant will have a score out of 8 (the total number of dietetic reviews over the 24-weeks), representing the number of reviews to have followed their dietary goals. See Objective 1.2 in Table 1.

##### 3.2.1.3. Dietary energy restriction

Estimated daily energy intake will be measured via a food diary for the 7 days leading into baseline, Week 24 (end of active interventions) and the 18-months follow-up. Participants will choose to complete the food diary either by a paper record or an online food diary equivalent (“Research Food Diary App”, Xyris Software Pty Ltd., Australia) depending on participant preference. Both these methods have been shown to result in similar nutrient intake estimates for participants following a weight loss intervention (27). Participants will be encouraged to complete the food diary in real time to minimize the potential of recall bias if diaries are completed retrospectively. The food diary will be entered into Foodworks 7 (Xyris Software Pty Ltd., Australia) to calculate total daily energy intake. The 24-week and 18-month average daily energy intake will be divided by the baseline average daily energy intake to provide an estimate of the percent change in energy intake achieved at each time-point. See Objective 1.3 in Table 1.

##### 3.2.1.4. Self-monitoring

Each SWIFt study participant will be provided with food for 2 days of the week totaling approximately 2,100 kJ/day (female) – 2,500 kJ/day (male). For the 5:2 intervention groups, the total energy content of the food to be provided is equivalent to the total energy intake permitted during fasting periods, and is designed to assist with adherence to the intervention. For participants in the CER group, the foods to be provided will be equitable in terms of what will be provided to all participants and, if these foods are consumed, should replace other foods in their diet (i.e., not increase energy intake and form part of their 20% energy restriction for the day). If a participant is unable to attend the clinic facility to collect food (e.g., due to COVID-19 travel restrictions), supermarket vouchers will be provided to allow a participant to buy the food items specified by the study dietitian. For all intervention groups, a food checklist for each week of the 24-week intervention will be provided to participants to note down the food provided, the date/time the food was consumed, the amount consumed (g/ml), and to note down other foods that were also consumed in addition to the study foods on that day. At each study visit, collection of the checklist will be recorded by the study dietitian (yes/no). Each participant will have a score out of 8 (the total number of dietetic reviews over the 24-weeks), representing whether checklists were collected at each visit. See Objective 1.4 in Table 1.

##### 3.2.1.5. Other engagement measures

Participant drop-out will be recorded, including date and reasons for drop-out (if specified). See Objective 1.5 in Table 1.

##### 3.2.1.6. Other SWIFt study quantitative data sources

Other SWIFt study data sources to be used for this evaluation have been described previously as part of the wider SWIFt study protocol (11) and include: weight at baseline, 24-weeks and 18-months; demographics and socioeconomic factors at baseline [age, sex, ethnicity (28), occupation, shift schedule, number of night shifts per fortnight, years in night shift]. See Objectives 2 and 3 in Table 1.

#### 3.2.2. Qualitative data sources

##### 3.2.2.1. Semi-structured interviews

In-depth, semi-structured interviews will be undertaken with participants from each dietary intervention. Three sets of interviews will be undertaken (at baseline, 24-weeks, and 18 months). Each interview is expected to be approximately 30–45 min in duration. An interview guide for each stage of interviews has been informed by existing dietary research in the shift working population (29, 30), the SWIFt pilot study, and the Theoretical Domains Framework (TDF) (see Supplementary material 1). The TDF provides a comprehensive set of determinants of behaviour grouped into constructs that have been derived from a review of relevant behaviour change theories (31). It has been successfully used for designing interview guides for previous evaluations of behaviour change interventions (32) and for analyzing interview data in a shift worker population (29). It is anticipated that interviews will occur either over the phone or via Zoom video-conference (Zoom Video Communications Inc., Version 5.13) at a time convenient to the study participant. The interviews will be recorded and transcribed verbatim (e.g., including ums, ahs, laughter and so on) by the main researcher (CD) or a transcription service. See Objectives 2 and 3 in Table 1.

The purpose of the baseline interview is to explore the motivations of participants for participating in the SWIFt study and perceived enablers or barriers to prior weight management. The interview will also identify what participants are hoping to achieve during the 24-week intervention.

The purpose of the 24-week (post-intervention) interview is to explore the participant’s experience of their allocated dietary intervention. In particular, to explore the perceptions of participants around reasons for engagement/non-engagement to the intervention, study factors thought to have assisted, and contextual factors (e.g., enablers and barriers at the individual, social, organisational, and wider environment) that have thought to influence engagement and weight change outcomes of the study.

The purpose of the 18-month interview is to explore the participant’s experience in following their allocated dietary approach without the support of the study intervention. This will explore the enablers and barriers to following their allocated dietary approach and weight management over this 12-month follow-up period.

##### 3.2.2.2. Longitudinal audio diaries (LADs)

A subset of participants will keep an approximate five-minute, fortnightly longitudinal audio diary (LAD) account of their experience of their dietary intervention over the 24-week period. LADs have been found to be a flexible and useful tool for enriching qualitative data collected on experiences over time that can be context specific (33). LADs allow participants to reflect on experiences at the time of the experience, rather than relying on recall as is typically the process of standard in-depth interviewing (33). In addition, it has been suggested that participants may more freely disclose matters of personal salience without the presence of a researcher (33).

The aim of the LADs is to capture the participant’s experience of their allocated dietary intervention each fortnight, in particular what has worked or not worked for aspects of food consumption and the enablers or barriers thought to have influenced this experience. Participants will be encouraged to use their mobile/smart phone to record their diary, or will be provided with an alternative option if preferred. An audio diary prompt sheet will be provided to participants with questions to consider (see Supplementary material 2), which has been designed in accordance with previous research using this method as an evaluation tool (34). A practice session with their mobile phone/recorder will be undertaken at the end of the first interview to ensure participants are able to use the technology. The audio diaries will be transcribed verbatim by the main researcher (CD) or a transcription service. After listening to the audio diary, if issues of concern are raised, such as participant discomfort, this will be discussed with the wider research team for appropriate next steps and escalation if required.

##### 3.2.2.3. Researcher reflexivity

Researcher reflexivity is an important component to allow for an awareness of how a researcher’s positionality may influence the research process, and to provide transparent and high-quality qualitative research (35, 36). The main researcher (CD) is an accredited practising dietitian undertaking this work as part of her PhD studies and has experience in both quantitative and qualitative research methods. CD will be involved in the wider SWIFt study as a researcher collecting data and as a study dietitian undertaking dietary consults with SWIFt participants. Reflexive diaries will be completed by the main researcher at key data points along the research process, including: after each participant interview, after reviewing participant LADs, and at each step of the data analysis process. Insights into this process will be discussed, as appropriate, at fortnightly meetings with the wider researcher team. This will allow documentation of key steps to the data collection and analysis process that can be reported as part of study results, adding transparency to the research process.

### 3.3. Data analysis

Quantitative data on participant engagement (see Objective 1 at Table 1) will be analyzed for frequency/count and proportions or as overall daily energy restriction as described above. Regression models will be used to examine associations between engagement measures, participant characteristics (sex, age, ethnicity, occupation, shift schedule, number of night shifts per fortnight, years in night shift), intervention group, and weight change. It is anticipated that general linear regression models will be used for dependent variables that are continuous data and generalized linear regression models will be used for dependent variables that are count data. Differences in drop-out/time to drop out between intervention groups will be examined via survival curve analysis.

Qualitative data includes interview transcripts and longitudinal audio diary transcripts and will be entered into NVivo (qualitative research computer software; Version 12) and analyzed using the five-steps of “framework analysis” (37, 38). In step one (*familiarization*), a subset of transcripts representing a mix of dietary intervention groups will be analyzed inductively by one researcher (CD) and reviewed by another researcher (SK) to identify influencing factors (intervention features in addition to enablers and barriers) for participant engagement for the allocated dietary intervention. Step two (*identifying a thematic framework*) involves developing an initial coding framework based on step one that details coding for the influencing factors for participant engagement. This step will also map enablers and barriers to the domains of the social-ecological model (SEM) (19) as a guide for developing themes and sub-themes. The SEM consists of five distinct domains (individual, social, organisational, community, public policy) and considers how these factors interact to influence health behaviours, enabling the development of strategies to improve behaviours. In step three (*indexing*), the framework will be used to code all data in NVivo. The Theoretical Domains Framework (TDF; described previously) (31) and the behaviour change taxonomy (BCT) (39) for intervention features, will also be used to map themes and sub-themes. The BCT is a set of 93 distinct behaviour change techniques that describe the components of an intervention thought to drive behaviour change (39). Where themes do not appear to fit within the mentioned frameworks, additional theoretical frameworks [e.g., Behaviour Change Wheel (BCW)] may be incorporated as necessary. In step four (*charting*), any differences between the dietary interventions and participant characteristics will be identified.

Finally, step five (*mapping and interpretation*) involves interpreting the findings based on existing literature. Merging of the qualitative and quantitative data (side-by-side comparison method) (40) will also be undertaken at this stage (see last two analysis steps at Figure 1). Qualitative themes will be used to help explain any associations found between engagement measures, participant characteristics, intervention group, and weight change from the quantitative data. This will be undertaken in both table and narrative form to assist in interpretation. Results from the different data sources will be integrated to confirm or refute the findings of each data source and to interrogate the validity of conclusions.

## 4. Discussion

Night shift workers are at greater risk for obesity, type 2 diabetes, and cardiovascular disease (2, 3). There is limited guidance on what dietary interventions may be useful for weight management in this population group (7–8). It has been suggested that both energy restriction and meal timing may be needed to address circadian misalignment and to result in effective weight loss (10, 11). To explore this concept, the Shifting Weight using Intermittent Fasting in night shift workers (SWIFt) study is a world-first, randomized controlled trial (RCT) that compares three weight-loss interventions (11). While the SWIFt RCT will determine the *effectiveness* of these dietary interventions for weight-loss, this mixed-methods evaluation will provide an important step in determining for who the SWIFt interventions work best for, what intervention features are important, and what external factors may need to be addressed to strengthen an approach. The findings from this mixed-methods evaluation will be useful for tailoring any future scalability of the SWIFt dietary weight-loss interventions for night-shift workers.

## 5. Ethics and dissemination

This mixed-methods evaluation protocol was approved by Monash Health Human Research Ethics Committee (RES 19-0000-462A) and registered with Monash University Human Research Ethics Committee. Ethical approval has also been obtained from the University of South Australia (HREC ID: 202379) and Ambulance Victoria Research Committee (R19-037). Consent to participate in both the quantitative and qualitative parts of the study was sought before data collection. Personal information (name and contact details) will be collected by study researchers and stored separately to research data in a password protected electronic database. Only the study’s researchers will have access to this information. All methods will be performed in accordance with the relevant guidelines and regulations of the approving ethics committees. Results from this evaluation will be disseminated via peer-reviewed journals, conference presentations, student theses, and presentations to interested workplaces that include shift workers.

## Ethics statement

The studies involving humans were approved by Monash Health Human Research Ethics Committee. The studies were conducted in accordance with the local legislation and institutional requirements. The participants provided their written informed consent to participate in this study.

## Author contributions

MB, CH, and JD were co-applicants on the original NHMRC grant application and were involved with the original design of the overall SWIFt trial that this mixed-methods evaluation is based on. CD, SK, MB, JD, and CH were involved in the design of this mixed-methods evaluation. CD is involved with study co-ordination and responsible for the day-to-day running of the evaluation, overall SWIFt trial, and including recruitment and sample collection for the qualitative data. SK is involved with review of qualitative data analysis as outlined in the methods section of this paper. JD and CH will assist CD with quantitative analyses and statistical interpretation of results. All authors contributed to the article and approved the submitted version.

## Funding

This project is funded by the National Health Medical Research Council [APP1159762]. The contents of the published material are solely the responsibility of Monash University and do not reflect the views of the Commonwealth.

## Conflict of interest

The authors declare that the research was conducted in the absence of any commercial or financial relationships that could be construed as a potential conflict of interest.

## Publisher’s note

All claims expressed in this article are solely those of the authors and do not necessarily represent those of their affiliated organizations, or those of the publisher, the editors and the reviewers. Any product that may be evaluated in this article, or claim that may be made by its manufacturer, is not guaranteed or endorsed by the publisher.
