# Supplementary material for: Evaluation of the “Shifting Weight using Intermittent Fasting in night-shift workers” weight loss interventions: a mixed-methods protocol
Source: Front Public Health. 2023 Sep 7;11:1228628. doi: 10.3389/fpubh.2023.1228628 (PMC10517326; doi:10.3389/fpubh.2023.1228628)
Supplement: Supplementary file 1 [file Table_1.DOCX]

**Appendix 1: Interview schedules**

**Interview Guide 1**

**SWIFt participant interview 1: Baseline Interview**

Thank you for agreeing to be interviewed about your experience of the SWIFt study. This is the first of three interviews. Overall, we are interested in hearing about how the SWIFt dietary strategy works (or does not work) for you, in particular what you may find easy or challenging in following the diet strategy. In this first interview, I am interested to hear about what motivated you to participate in the SWIFt study, what you feel currently impacts your diet or eating patterns or habits and your thoughts on your allocated dietary strategy and any other diet strategies you have tried in the past.

Just as a reminder, the interview will be audio-recorded, but I’d like to assure you that all comments will be de-identified using a unique code and data will be stored securely following Monash privacy and data storage policies.

Do you have any questions before we begin?

| Questions and prompts: Baseline interview | Prompts | Purpose/TDF* components |
| --- | --- | --- |
| Please tell me a bit about yourself and your shift-work role |  | Introduction/rapport |
| What motivated you to participate in our study? | What are you hoping to achieve out of participating in this study?  How do you think the study will help you achieve this? | Intensions  Goals |
| The next set of questions are about your current eating patterns or habits: | | |
| How would you describe your current eating patterns? | How do you feel it affects your overall health? | Knowledge  Beliefs about consequences |
| In what way does night shift work affect your eating patterns?   - During work? - At home? | During work - If not mentioned, could probe for facilities, food availability, workplace support, scheduling/breaks, lack of routine, food as reward, time, fatigue, what others do)  At home - If not mentioned, could probe for time, fatigue | Environmental/social influences |
| Are there any other factors apart from shift work that affect your eating patterns? |  | Environmental/social influences |
| The next set of questions are about the SWIFt dietary strategy you have been allocated to, the _____ diet strategy. | | |
| What does the ______ diet strategy mean to you? |  | Knowledge |
| How do you feel about your allocated diet strategy? |  | Optimism  Beliefs about consequences |
| Have you tried this diet strategy before?   - Can you tell me about this experience? - What worked/did not work for you? How?   Have you tried any other weight loss strategies before?   - Can you tell me about this experience? - What worked/did not work for you? How? |  | Beliefs about capabilities  Environmental/social influences |
| Are there any other matters about shift work and your eating patterns that you would like to mention? |  | All domains |
| Provide information on longitudinal audio diaries if applicable |  |  |

* Theoretical Domains Framework (TDF; Atkins, 2017)

Atkins, L., Francis, J., Islam, R., O’Connor, D., Patey, A., Ivers, N., ... & Lawton, R. (2017). A guide to using the Theoretical Domains Framework of behaviour change to investigate implementation problems. *Implementation Science*, *12*(1), 77.

**Interview Guide 2**

**SWIFt participant interview 2: 6-months**

Once again, thank you for agreeing to be interviewed about your experience during the SWIFt study. This is the second of three interviews. Overall, we are interested in hearing about how the SWIFt dietary strategy worked (or did not work) for you, in particular what you found easy or challenging in following the diet strategy. In this second interview, I am interested to hear about your experience of following your SWIFt ____ diet strategy over the past 6 months, and what helped or did not help you to follow the dietary strategy.

Just as a reminder, the interview will be audio-recorded, but I’d like to assure you that all comments will be de-identified using a unique code and data will be stored securely following Monash privacy and data storage policies.

Do you have any questions before we begin?

| Questions and prompts: 6-month interview | Prompts | Purpose/TDF* components | |
| --- | --- | --- | --- |
| The first set of questions are about your overall experience of the ____SWIFT diet strategy. | | | |
| Please briefly tell me about the SWIFt diet strategy you were allocated to over the past 6 months   - How would you describe your motivation to follow your SWIFt diet over the past 6-months? | What affected your motivation? | Introduction/rapport  Optimism  Beliefs about consequences | |
| Please describe what it was like in the first few weeks of following the diet? | What did you like the best? Why?  What didn’t you like about this diet? Why? | All domains | |
| Following on from the last question, can you tell me about your experience for the rest of the 24-weeks of the dietary strategy?   - How did the experience compare to other diets you have tried in the past (if applicable)? |  | All domains | |
| What was it like being on night shift while following the diet?   - What was helpful about following the diet during night shift? How? - Tell me about some challenges (if any) you have faced being on this diet whilst on night shift? - What strategies did you use to help you overcome these challenges? How did you develop these strategies? | What was it like ‘fasting’ during this time (5:2N only)?  In what way (if any) do you think shift work influenced your ability to make changes to your diet? | All domains | |
| Can you tell me what was it like on your non-night shift days while following the diet?   - What was helpful about following the diet during non-night shift days? How? - Tell me about some challenges (if any) you have faced being on this diet during your non-night shift days? - What strategies did you use to help you overcome these challenges? How did you develop these strategies? |  | All domains | |
| What do you feel you have got out of participating in the SWIFt study? | How did the study lead to this? |  | |
| How do you feel about continuing the ____ SWIFt diet strategy into the future?  Why would you/would you not like to continue? |  | Skills/Intensions/Goals  Environmental/social influences | |
| The next set of questions are about the supports that were provided to you while following the ___ SWIFt diet strategy. Supports include things such as the food provided, dietetic consultations and monitoring your progress. | | |  |
| Since participating in the study, we have advised you to change some of your usual eating habits.   - What worked? How? - What did not work? How? - How would you describe your ability to follow the suggested changes? - How easy was it to understand the instructions? - How easy was it to follow the instructions? - How did your shift work influence your ability make the suggested changes? | [review responses to baseline interview to see whether previous shift work barriers are covered] | Knowledge  Skills  Goals  Environmental/social influences | |
| Thinking back to the supports I described earlier such as the provision of food and dietetic consultations, can you tell me how you feel they contributed to your experience over the past 6-months. | How important were the provided meals? Timing of provided meals?  How do you feel about the initial dietetic consult?  How do you feel about the regular consults with your dietitian?  What influenced whether you caught up regularly with your dietitian?  What did you learn about yourself and your dietary habits during the 6-months? How did this happen? | Knowledge  Skills  Behavioural regulation (self-monitoring)  Goals  Intensions  Environmental/social influences | |
| How did you feel monitoring your progress contributed to your experience during the 6 months? | Measures at clinic?  Weight monitoring via electronic scales?  Do you find that it encouraged you or discouraged you to keep following this diet? How? | Behavioural regulation (self-monitoring) | |
| The last set of questions are about changes we could make to the _____SWIFT diet strategy to make it work better. | | | |
| What aspects of this research study do you think could be barriers for you or others to participate? | For example, having to drive here regularly? Zoom? Or anything else?  What would you do to make it easier? | Environmental/social influences | |
| What suggestions do you have (if any) to improve the ____ SWIFt diet strategy? | Any changes you would make in terms of the food?  Dietary consults?  How about the timing or frequency of the fasting? How do you feel about it? (5:2 only) | All domains | |
| Insert here any other questions based on review of the participant’s longitudinal audio diary (LAD) (if relevant) |  |  | |
| Are there any other matters about your SWIFt diet over the past 6-months that you would like to mention? |  |  | |

* Theoretical Domains Framework (TDF; Atkins, 2017)

Atkins, L., Francis, J., Islam, R., O’Connor, D., Patey, A., Ivers, N., ... & Lawton, R. (2017). A guide to using the Theoretical Domains Framework of behaviour change to investigate implementation problems. *Implementation Science*, *12*(1), 77.

**Interview Guide 3**

**SWIFt participant interview 3: 18-months**

Once again, thank you for agreeing to be interviewed about your experience during the SWIFt study. This is the last of three interviews. Overall, we are interested in hearing about how the SWIFt dietary strategy worked (or did not work) for you, in particular what you found easy or challenging in following the diet strategy. In this second interview, I am interested to hear about whether you continued to follow your _____ SWIFt diet strategy over the past 6 months. I am also interested to her about your experience without the support of the study over the past 6 months, and what helped or did not help you during this period.

Just as a reminder, the interview will be audio-recorded, but I’d like to assure you that all comments will be de-identified using a unique code and data will be stored securely following Monash privacy and data storage policies.

Do you have any questions before we begin?

| Questions and prompts: 6-months post intervention interview | Prompts | Purpose/TDF* components |
| --- | --- | --- |
| Please tell me about what your diet has been like over the past 6-months.   - What has helped (or not helped) you with managing your weight? - What (if any) challenges have you faced? What did you do when faced with these challenges? |  | Introduction/rapport  All domains |
| Have you tried following the SWIFt diet strategy over the past 6-months? Why/why not?   - What has worked? How? - What has not worked? How? - What aspects of your shift work have helped/not helped? |  | All domains  Environmental influences |
| What aspects (if any) of the initial 6-months of the SWIFt study have helped you over the past 6-months? How? | What aspects of your previous dietary consults (if any) have helped? | Skills/Intensions/Goals |
| Have you tried any other weight management approaches over the past 12-months? Why/why not?   - What has worked? How? - What has not worked? How? |  | All domains |
| What do you think would help you maintain your weight into the future? How? |  | All domains |
| Is there anything else you would like to mention about your diet over the past 6-months? |  |  |

* Theoretical Domains Framework (TDF; Atkins, 2017)

Atkins, L., Francis, J., Islam, R., O’Connor, D., Patey, A., Ivers, N., ... & Lawton, R. (2017). A guide to using the Theoretical Domains Framework of behaviour change to investigate implementation problems. *Implementation Science*, *12*(1), 77.
